# Ultrafast giant magnetic cooling effect in ferromagnetic Co/Pt multilayers

**DOI:** 10.1038/s41467-017-00816-w

**Published:** 2017-10-06

**Authors:** Je-Ho Shim, Akbar Ali Syed, Chul-Hoon Kim, Kyung Min Lee, Seung-Young Park, Jong-Ryul Jeong, Dong-Hyun Kim, Dong Eon Kim

**Affiliations:** 1 Department of Physics, Chungbuk National University, Cheongju, 361-763 South Korea; 2 Department of Physics and Center for Attosecond Science and Technology, POSTECH, Pohang, 790-784 South Korea; 3 Max Planck Center for Attosecond Science, Max Planck POSTECH/KOREA Research Initiative, Pohang, 790-784 South Korea; 4 Department of Advanced Materials Chemistry, Korea University, Sejong, 339-700 South Korea; 5 Department of Material Science and Engineering and Graduate School of Energy Science and Technology, Chungnam National University, Daejeon, 305-764 South Korea; 6 Spin Engineering Physics Team, Korea Basic Science Institute, Daejeon, 305-806 South Korea

## Abstract

The magnetic cooling effect originates from a large change in entropy by the forced magnetization alignment, which has long been considered to be utilized as an alternative environment-friendly cooling technology compared to conventional refrigeration. However, an ultimate timescale of the magnetic cooling effect has never been studied yet. Here, we report that a giant magnetic cooling (up to 200 K) phenomenon exists in the Co/Pt nano-multilayers on a femtosecond timescale during the photoinduced demagnetization and remagnetization, where the disordered spins are more rapidly aligned, and thus magnetically cooled, by the external magnetic field via the lattice-spin interaction in the multilayer system. These findings were obtained by the extensive analysis of time-resolved magneto-optical responses with systematic variation of laser fluence as well as external field strength and direction. Ultrafast giant magnetic cooling observed in the present study can enable a new avenue to the realization of ultrafast magnetic devices.

## Abstract (web summary)

The forced alignment of magnetic moments leads to a large change in entropy, which can be used to reduce the temperature of a material. Here, the authors show that this magnetic cooling effect occurs on a femtosecond time scale in cobalt–platinum nano-multilayers.

## Introduction

Since the discovery of the giant magnetocaloric effect^1, 2^, there has been considerable interest in understanding the fundamental magnetic cooling mechanism^3–8^. The core process of the magnetic cooling effect (MCE) is that a material temperature is reduced as a result of the forced alignment of the magnetic moments by an external magnetic field. So far, most studies on the MCE have been carried out on bulk oxide materials with specific structures, such as perovskite or Heusler type, because the maximal MCE is normally observed around the structural and/or magnetic phase transitions^3–6^. Recently, MCE has also been observed for nanostructured films, such as La_0.7_Ca_0.3_MnO_3_ film^7^ and vanadium oxide superlattice films on graphene^8^, where the underlying mechanism for the observed MCE results is still based on a structural phase transition.

Because the MCE is the result of forced magnetization alignment by an external field, the question of how fast the MCE can be realized naturally arises. The answer to the question must involve the dynamics of demagnetization and subsequent remagnetization of magnetic materials. Over the past two decades, an ultrafast photoinduced demagnetization-remagnetization phenomenon via the time-resolved magneto-optical Kerr effect (TR-MOKE) has been investigated intensively since the seminal work by Beaurepaire et al.^9^. The ultrafast demagnetization and remagnetization process of the net magnetic moments triggered by light pulses has inspired substantial interest in the ultrafast interaction dynamics among spin, electron, lattice and photon on a femtosecond timescale. Quite recently, femtoseond laser heating was reported to play an important role in determining the ultrafast photoinduced demagnetization and remagnetization phenomenon for ferrimagnetic GdFeCo^10, 11^ and ferromagnetic FePt^12^. Therefore, confirmation of the existence of the MCE during femtosecond laser heating becomes essential for further understanding the relevant spin, electron and lattice dynamics. On the other hand, no work has been done to answer the question as to how fast MCE can be realized if the MCE exists during the photoinduced demagnetization and remagnetization process. To reveal the existence of MCE on the ultrafast timescale, it is important to track the spin, electron and lattice temperatures separately.

In the case of ferromagnetic metallic films, the characteristic demagnetization time is known phenomenologically to be shorter than the electron relaxation time^13, 14^ for most systems, so that the spin temperature is assumed to instantly follow the electron temperature^14–17^. However, the assumption of the same temperatures for spin and electron in ferromagnetic metallic film systems needs to be clarified since it has been reported that there is a substantial temperature difference between spin and electron for a half-metallic system^18^.

In the following, we report our experimental demonstration of the existence of the MCE phenomenon on a femtosecond timescale. TR-MOKE experiment has been carried out for ferromagnetic Co/Pt multilayers, where the 3-temperature model (3TM)^9^ was applied to separately track the temperatures of spin, electron and lattice after being excited by femtosecond laser. From our systematic investigation with variation of external magnetic field strengths as well as pump fluences, it is concluded that the separated temperatures of spin, electron, and lattice and independent cooling of spin sub-system by the external magnetic field on a sub-picosecond timescale is considered to be a main origin of the ultrafast giant MCE. Ultrafast giant MCE by the forced alignment of magnetization under external magnetic fields is found to be a universal phenomena, observed for all Co/Pt multilayers with repeat number of 5, 10 and 15. The existence of the giant MCE was also confirmed by exponential fitting of the relaxation behavior during the remagnetization of the Co/Pt multilayer systems.

## Results

### TR-MOKE measurement

**Fig. 1:**
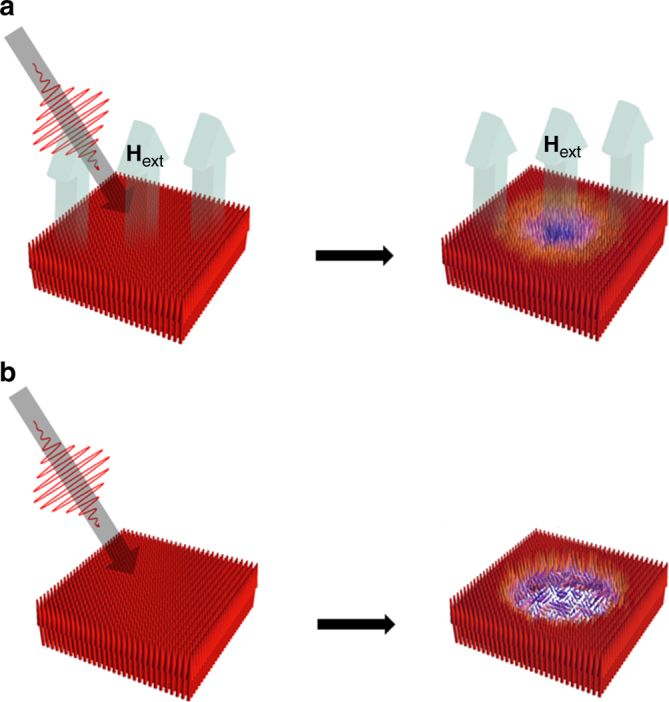
Schematic diagram of the ultrafast magnetic cooling effect. **a** Upwardly saturated initial spin moments under an external magnetic field (**H**_ext_) before a laser pulse excites the sample (left). Laser pulse propagation direction is represented by gray arrow. Demagnetized state after a laser pulse hits the sample under **H**_ext_ (right). **b** Upwardly saturated initial spin moments before a laser pulse excites the sample (left). Demagnetized state after a laser pulse hits the sample under zero external magnetic field (right)

Figure 1 illustrates how a spin state demagnetized by a femtosecond laser pulse might be forced to be aligned under an external magnetic field, leading to a decrease in the effective spin temperature (Fig. 1a) compared to the case of demagnetization under a zero external field (Fig. 1b). To carry out a systematic investigation of this magnetic cooling phenomenon on a femtosecond time scale, it is important to have material parameters controlled in a desired manner. A ferromagnetic metallic multilayer provides an interesting playground to explore the above mechanism because important magnetic properties, such as the perpendicular magnetic anisotropy (PMA) and saturation magnetization, are engineered easily by controlling the multilayer parameters, such as the repeat number and sublayer thickness^19–23^. Another important feature of the ferromagnetic metallic multilayer is that there is no giant MCE originating from the oxide-related structural change. Therefore, if the MCE is measured in the ferromagnetic metallic multilayers, it must have purely originated from the forced alignment of magnetic moments by an external field.

We have measured TR-MOKE signal both with modulating pump-beam and with modulating probe-beam by a mechanical chopper during the stroboscopic measurement, where the systematic comparison is fully discussed in the Supplementary Notes 2–5. While the TR-MOKE signal measured with probe-beam modulation simply detects a signal proportional to a magnetization with an improved signal-to-noise ratio^9, 24, 25^, the TR-MOKE signal measured with pump-beam modulation selectively detects a signal proportional to the change in the magnetization induced by the pump-beam rather than the magnetization itself^9, 26–31^. It should be mentioned that the sum of the pump- and probe-beam modulated loops is to be invariant over all negative and positive time delays and confirmed experiments (Supplementary Note 4). Since the summed loop is constant, TR-MOKE signal either with pump- or with probe-beam modulation can be analyzed without loss of generality. Here, we focus on the evolution of hysteresis loops measured with pump-beam modulation during the photoinduced demagnetization-remagnetization process.

**Fig. 2:**
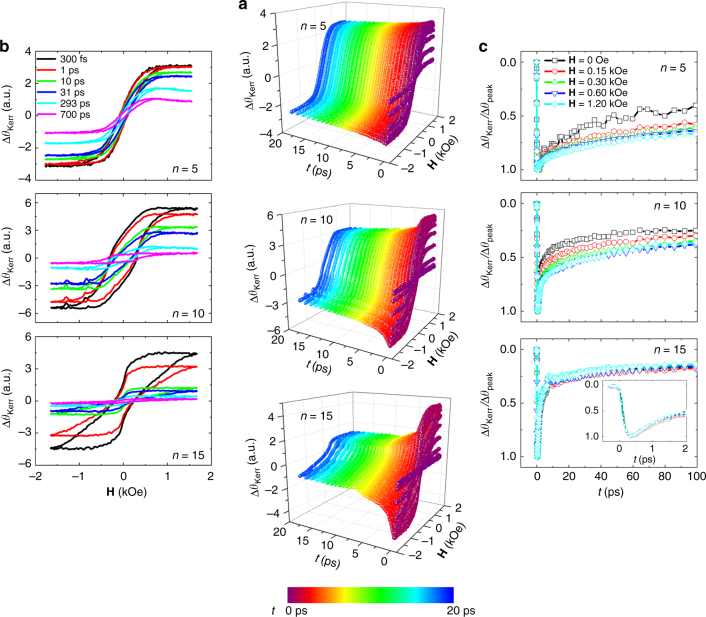
Time-resolved magneto-optical Kerr effect signals. **a** 3D-map of TR-MOKE of [Co/Pt]_n_ with *n* = 5, 10, and 15. The color code on the bottom indicates a stroboscopically elapsed time from 0 to 40 ps. **b** Time-dependent *∆θ*_Kerr_ hysteresis loops measured by pump-beam modulation at different delays from 300 fs to 700 ps for *n* = 5, 10 and 15. (**c**) Field-dependent normalized TR-MOKE signal *(∆θ*_Kerr_/*∆θ*_peak_) for *n* = 5, 10 and 15. The inset in the bottom shows a TR-MOKE signal within 2 ps

In Fig. 2, the TR-MOKE signals during the demagnetization and remagnetization are also plotted with respect to the field (**H**) and time (*t*). **H** was applied with an angle of 23° from the film normal. The MOKE signal vs. **H** (Fig. 2b) provides the time-resolved magnetic hysteresis loop at a specific delay and the temporal change in the MOKE signal (Fig. 2c) provides information at a specific applied field. The color code of Fig. 2a (purple to blue) represents the delay time between the time zero and 20 ps. Note also that the TR-MOKE feature becomes different for different repeat numbers. Generally, hysteresis loops represented by *∆θ*_Kerr_ (Fig. 2b) exhibit a rapid increase in the loop height until *t* ~ 300 fs and a slow decrease thereafter for all the films. On the other hand, the detailed relaxation behavior for *t* > 300 fs is substantially different for different repeat numbers. The TR-MOKE signals shown in Fig. 2c exhibit the typical behavior of the rapid demagnetization around 300 fs, which is followed by slow relaxation on a long time scale lasting for up to few hundreds of picoseconds, leading to remagnetization characteristic of the type-I ferromagnetic system^14^. The inset in the bottom figure of Fig. 2c shows a TR-MOKE signal at various fields during the initial 2 ps, where the demagnetization reaches its peak ~*t* = 300 fs within the error range.

Interestingly, the photoinduced demagnetization-remagnetization phenomena also become different for different repeat numbers. For the multilayer film with *n* = 15, a recovery of more than 50% was observed after just several picoseconds, while a slower recovery was observed as n decreases. Attention should be paid to the fact that the photoinduced demagnetization–remagnetization process becomes quite field-dependent in the case of *n* = 5 and 10, whereas the process becomes less dependent on the external magnetic field strength in the case of *n* = 15.

### 3TM analysis

To understand the observed features, the TR-MOKE hysteresis loops were analyzed in full details. Considering that the present results were obtained with the pump-beam modulation, TR-MOKE signals measured in the present setup are not proportional to the absolute magnetization value but to the change in the magnetization induced by the pump laser pulse. Therefore, the hysteresis loops in Fig. 2b are not conventional magnetic hysteresis loops, providing the macroscopic parameters, such as remanent magnetization (*M*_R_), saturation magnetization (*M*_S_). Instead, they indicate how much the corresponding physical quantities are changed; i.e., ∆*M*_R_ and ∆*M*_S_, respectively.

For the measured TR-MOKE hysteresis loops, under 3TM^9^, the following equations were used to fit the experimental data:

1 $$\begin{array}{l}\\ {C_{\rm{e}}}({T_{\rm{e}}})\frac{{{\rm d}{T_{\rm{e}}}}}{{{\rm d}t}}{\rm{ = }} - {G_{{\rm{el}}}}({T_{\rm{e}}} - {T_{\rm{l}}}) - {G_{{\rm{es}}}}({T_{\rm{e}}} - {T_{\rm{s}}}) + P(t),\\ \\ {C_{\rm{l}}}({T_{\rm{l}}})\frac{{{\rm{d}}{T_{\rm{l}}}}}{{{\rm{d}}t}}{\rm{ = }} - {G_{{\rm{el}}}}({T_{\rm{l}}} - {T_{\rm{e}}}) - {G_{{\rm{ls}}}}({T_{\rm{l}}} - {T_{\rm{s}}}) - {K_{\rm{l}}}{({T_{\rm{l}}} - 300)^3},\\ \\ {C_{\rm{s}}}({T_{\rm{s}}})\frac{{{\rm{d}}{T_{\rm{s}}}}}{{{\rm{d}}t}}{\rm{ = }} - {G_{{\rm{es}}}}({T_{\rm{s}}} - {T_{\rm{e}}}) - {G_{{\rm{ls}}}}({T_{\rm{s}}} - {T_{\rm{l}}}),\\ \end{array}$$

where *T*_e_, *T*_s_ and *T*_l_ are the electron, spin and lattice temperatures, respectively. *C*_e_, *C*_s_ and *C*_l_ are the specific heats of the electron, spin and lattice, respectively. *G*_el_ is the electron–lattice interaction parameter. *G*_es_ and *G*_ls_ are the electron–spin and lattice–spin interaction parameter, respectively. *P*(*t*) is a laser source with a Gaussian temporal profile. The term containing *K*_*l*_ is a lattice thermal diffusion that is modeled to be proportional to the third power of the temperature difference^32^. In most cases, the thermal diffusion term could be neglected within a few tens of picoseconds in the case of an ultrafast demagnetization–remagnetization process of ferromagnetic metallic films^9, 33^. In addition, we also confirmed that a variation in the power of the thermal diffusion term does not modify the fitting result significantly, particularly in time scales less than *t* < 40 ps. The results of 3TM were applied to match the observed quantity of the normalized MOKE signal (|*∆θ*_Kerr_|/|*∆θ*_peak_|), using $\Delta M\, \propto 1 - \frac{{\sqrt {1 - {{({T_S}{\rm{/}}{T_C})}^2}} - \sqrt {1 - {{(T_S^{{\rm{peak}}}{\rm{/}}{T_C})}^2}} }}{{\sqrt {1 - {{(300{\rm{K/}}{T_C})}^2}} - \sqrt {1 - {{(T_S^{{\rm{peak}}}{\rm{/}}{T_C})}^2}} }}$ (ref. ^33^). A conventional 2-temperature model (2TM) was initially attempted, resulting in poor fittings, particularly under cycling external fields. A detailed comparison of fitting results by 3TM and 2TM is described in the Supplementary Note 6. A comparison between the experimental and 3TM results was carefully made, resulting in excellent agreement, as demonstrated in Supplementary Figs 8–10. Table 1 lists the fitting parameters.

**Table 1:** List of fitting parameters with variation of pump fluences and external field angles

	9.9 mJ cm^−2^ (*θ*_H_ = 0°)	13.2 mJ cm^−2^ (*θ*_H_ = 0°)	16.5 mJ cm^−2^ (*θ*_H_ = 0°)	13.2 mJ cm^−2^ (*θ*_H_ = 23°)
*[Co/Pt]*_*5*_ *multilayer*				
*G*_el_ (10^17^ W m^−3^ K^−1^)	2.00	1.00	1.00	0.40
*G*_es_ (10^17^ W m^−3^ K^−1^)	30.0	10.0	5.50	10.0
*G*_ls_ (10^17^ W m^−3^ K^−1^) [**H** = 0 Oe]	1.50	0.40	0.20	0.40
*G*_ls_ (10^17^ W m^−3^ K^−1^) [**H** = 1.70 kOe]	11.4	3.00	1.32	2.20
*[Co/Pt]*_*15*_ *multilayer*				
*G*_el_ (10^17^ W m^−3^ K^−1^)	6.74	6.00	5.30	6.74
*G*_es_ (10^17^ W m^−3^ K^−1^)	30.0	2.00	1.50	30.0
*G*_ls_ (10^17^ W m^−3^K^−1^) [**H** = 0 Oe]	0.60	0.30	0.25	2.70
*G*_ls_ (10^17^ W m^−3^ K^−1^) [**H** = 1.70 kOe]	1.60	0.90	0.75	3.30

**Fig. 3:**
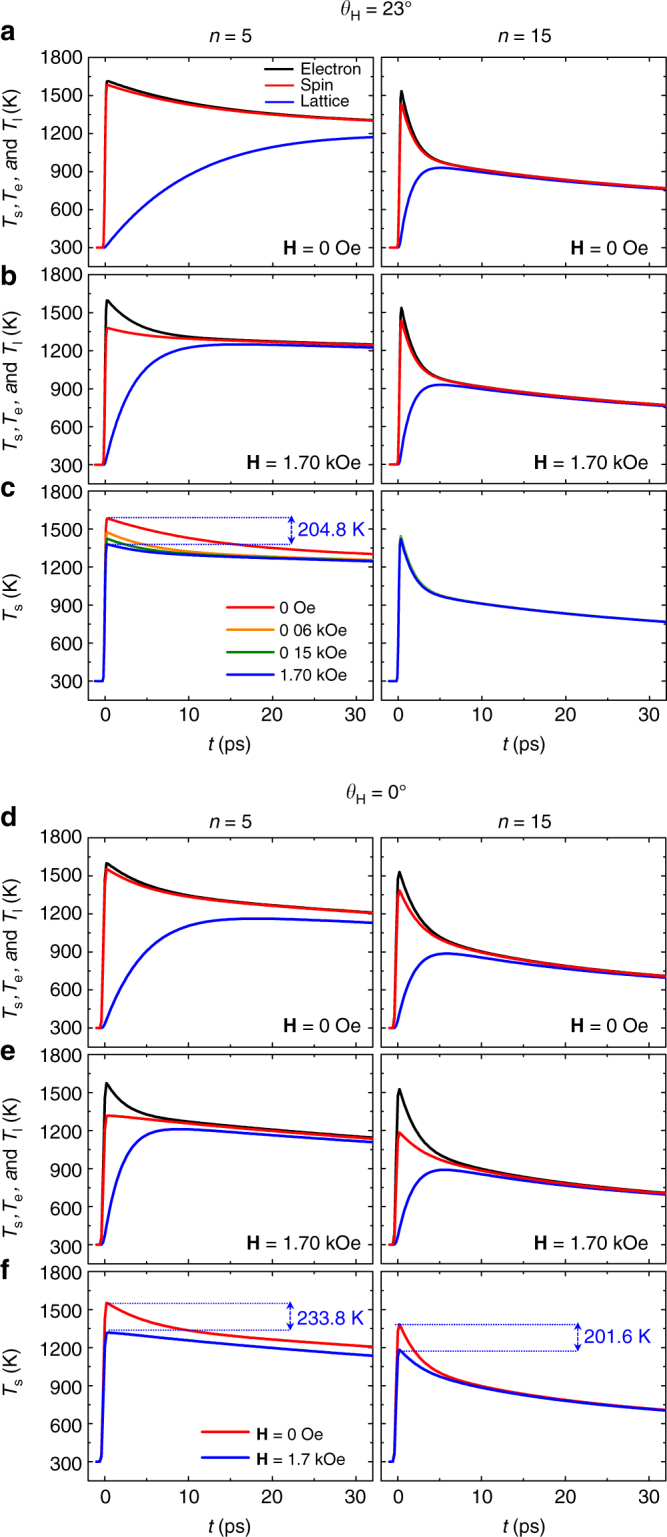
Temperatures of electron, spin and lattice. Temporal behavior of *T*_s_ (red), *T*_e_ (black) and *T*_l_ (blue) determined from 3TM analysis for *n* = 5 (left) and 15 (right) with *θ*_H_ = 23° **a** under a zero field and **b** external field of 1.70 kOe. **c** *T*_s_ with respect to the delay for various fields of **H** between 0 and 1.70 kOe. *T*_s_ (red), *T*_e_ (black) and *T*_l_ (blue) determined from 3TM analysis for *n* = 5 (left) and 15 (right) with *θ*_H_ = 0° (**d**) under a zero field and (**e**) external field of 1.70 kOe. (**f**) *T*_s_ with respect to the delay for **H** = 0 and 1.70 kOe

The excellent fitting in the case of the external field angle *θ*_H_ = 23° provides information on the temporal behaviors of *T*_s_, *T*_e_ and *T*_l_, as plotted in Fig. 3 with the parameters listed in Table 1. In case of *n* = 15, the equilibrium temperature, *T*_eq_, where *T*_e_ becomes equal to *T*_l_ for the first time is ~ 937 K at *t* = 8.5 ps under zero field. In the case of *n* = 5, *T*_eq_ ~ 1232 K at *t* = 65 ps under a zero field (not shown). As *T*_s_ is related to the magnetization, which is a magnetic ordering parameter, the lower *T*_s_ indicates a higher degree of magnetization (or equivalently larger remagnetization). The fitting results (Table 1) indicate that *G*_el_ increases in proportion to *n*, as previously reported for the case of 2TM under no external field^34^. The increased *G*_el_ indicates an increase in the electron–lattice interaction, suggesting that the remagnetization converging to the equilibrium becomes faster. This interesting feature that the remagnetization becomes faster and larger with increasing *n* possibly implies the controllability of the electron–lattice interaction with *n*.

The behaviors of *T*_s_, *T*_e_ and *T*_l_ with the variation of the applied magnetic field strength were examined. In Fig. 3, *T*_s_, *T*_e_ and *T*_l_ under 1.70 kOe was also plotted for *n* = 5 and 15. The equilibrium time (*t*_eq_) required for *T*_s_, *T*_e_ and *T*_l_ to reach *T*_eq_ was approximately several tens of picoseconds for *n* = 5, whereas *t*_eq_ is approximately a few picoseconds for *n* = 15. The 3TM fitting indicated that *G*_el_ increases with *n*. With increasing *G*_el_, *t*_eq_ should be shortened. Therefore, *T*_s_ reaches *T*_eq_ more rapidly in the case of *n* = 15 than for the case of *n* = 5.

**Fig. 4:**
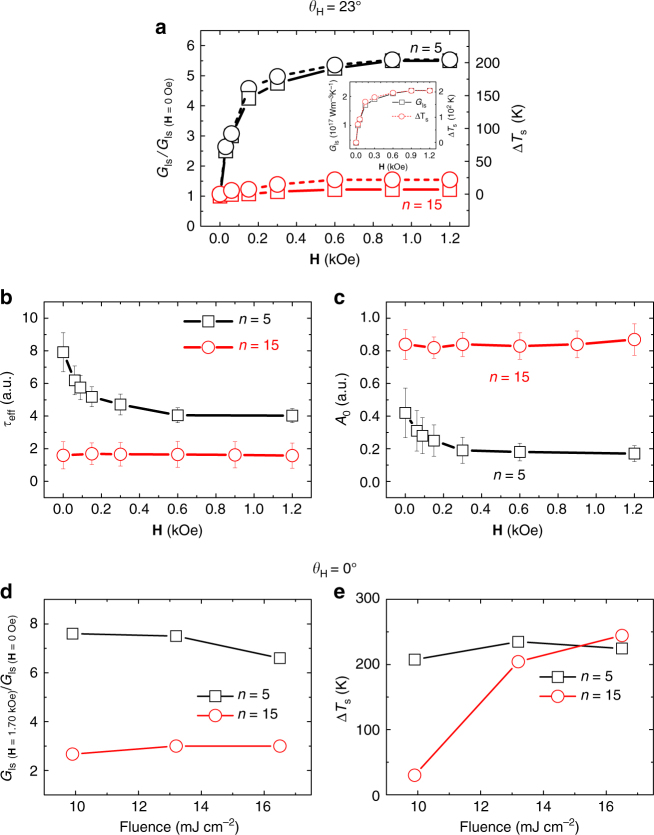
Field- and fluence-dependent behavior. At *θ*_H_ = 23°, **a** Field-dependent *G*_ls_/*G*_ls(**H**=0)_ (open square solid line) and *∆T*_s_ (open circle dotted line) for *n* = 5 (black) and 15 (red). Fitted values of *τ*_eff_ **b** and *A*_0_ **c** under various fields for *n* = 5 (open square) and 15 (open circle) samples. Fluence-dependency at *θ*_H_ = 0° for **d** *G*_ls(**H**__=__1.70__kOe)_/*G*_ls(**H**__=__0)_ and **e** *∆T*_s_ for *n* = 5 (open square) and 15 (open circle) samples

Attention should be drawn to another interesting point that in the case of *n* = 5, under an applied field of 1.70 kOe, *t*_eq_ becomes significantly shorter, while there is little shortening for *t*_eq_ in the case of *n* = 15 as shown in Fig. 3. In other words, under a zero field, *T*_s_ follows *T*_e_ almost instantaneously on a demagnetization–remagnetization time scale, whereas under an external field, *T*_s_ follows *T*_e_ but with a delay: a clear separation of spin and electron temperatures was observed in the left part of Fig. 3b, c. As plotted in Fig. 3c, the difference in *T*_s_ between **H** = 0 and 1.70 kOe becomes largest (204.8 K) at *t* = 300 fs and decreases thereafter in the case of *n* = 5. Note that *T*_e_ is not changed significantly by **H** but only *T*_s_ is lowered substantially under an external magnetic field at the initial phase (*t* < 20 ps), clearly indicating that, there exists a MCE on the sub-picosecond timescale, as predicted in Fig. 1. The cooling of 204.8 K, as shown in Fig. 3c, is enormous. Figure 4a shows the amount of cooling (∆*T*_s_) under various **H** for *n* = 5 and 15 samples.

### Ultrafast magnetic cooling effect

The observed ultrafast giant MCE behavior is compared to a static MCE, which was analyzed from a field- and temperature-dependent magnetization behavior measured by means of superconducting quantum interference device. The specific heat of the samples was also measured by differential scanning calorimeter (DSC). The static MCE results are shown in the Supplementary Note 7. Total heat capacity values experimentally determined by DSC are 1.85 × 10^6^ J m^−3^ K^−1^ (*n* = 5) and 2.15 × 10^6^ J m^−3^ K^−1^ (*n* = 15). The total heat capacity values measured by DSC are in a good agreement with the total sum of the heat capacities (*C*_l_ *+* *C*_e_ *+* *C*_s_) from the 3TM fitting as in Table 1, 1.63 × 10^6^ J m^−3^ K^−1^ (*n* = 5) and 3.19 × 10^6^ J m^−3^ K^−1^ (*n* = 15), respectively.

The cooling temperature *(∆T*) in the conventional static MCE is given as follows:

2 $$\Delta T{\rm{ = - }}\mu \mathop {\int}\limits_0^{{H_{{\rm{Sat}}}}} {\frac{T}{C}} \left( {\frac{{\partial M}}{{\partial T}}} \right){\rm{d}}H,$$

where *μ* is permeability and *C* is a total specific heat^1^. A huge cooling (large *∆T*) implies the drastic reduction of specific heat. In a static equilibrium case, a heat capacity with contributions from lattice, electron and spin will mostly increase if the system temperature increases, or at most, it should not decrease. The cooling temperature from the static MCE is found to be only 27 and 14 mK for *n* = 5 and 15 samples, substantially smaller than the cooling temperatures from the ultrafast MCE. Thus, the huge cooling observed in the present study cannot be explained within the context of the equilibrium MCE. We conclude that the observed giant MCE on the femtosecond timescale is fundamentally originated from the ultrafast non-equilibrium phenomenon. Unlike the equilibrium, spin, electron and lattice sub-system could be separated in a non-equilibrium and each sub-system independently behaves with separated value of specific heat on a femtosecond timescale. Thus, the temperature of the spin system, thermally separated from electron and lattice on ultrafast time scale, might be independently reduced with much smaller *C*_s_ value: the replacement of *C* with *C*_s_ in Eq. (1) leads to a huge *∆T*_s_.

Considering that the MCE is mainly a result of a forced alignment of spin, the observed ultrafast giant MCE should depend more sensitively on the strength of the aligning field. The aligning effective field is a vector sum of the external field (**H**_ext_) and the anisotropy field (**H**_k_) of the sample. The measured **H**_k_ was 12.6 and 6.6 kOe for *n* = 5 and 15 sample, respectively, as in the Supplementary Note 8. We have repeated the same MCE experiment and the 3TM analysis with an external field direction normal to the film *(θ*_H_ = 0°*)*, where the effective field is expected to be stronger compared to the case of *θ*_H_ = 23°. The TR-MOKE signal is found to be fitted well again by the 3TM (see Supplementary Fig. 10), resulting in the temperature variation shown in Fig. 3d–f. Interestingly, the substantial MCE is now observable even for *n* = 15 sample. The 3TM analysis implies that initially (<5 ps) spin temperature is clearly separated and independently cooled by the **H**_ext_ on the ultrafast timescale before the temperatures reach equilibrium. Note that the time *(t*_eq_) required to reach equilibrium becomes shorter for the case of *n* = 15 than for the case of *n* = 5, as previously observed for the case of *θ*_H_ = 23°. It should be reminded that the resulting MCE is represented not by the value of *t*_eq_ itself but by the change of *t*_eq_ under external fields. *t*_eq_ is dramatically shortened again from 121 to 33.8 ps in case of *n* = 5, while, to a lesser degree, from 25 to 15 ps in case of *n* = 15. This is not so surprising since the ultrafast MCE due to the forced spin alignment under external fields should be rather a universal phenomenon.

We have carried out further experiments with the variation of pump fluence from 9.9 to 16.5 mJ cm^−2^. The experiments have been carried out with external fields applied normal to the films (*θ*_H_ = 0°). The 3TM analysis of fluence-dependent TR-MOKE signals is described in Supplementary Fig. 10. The 3TM still fits to the data very well, where we used the same values for heat capacities of spin, electron and lattice as previous ones, while freeing the *G*_el_, *G*_es_ and *G*_ls_ parameters. For the case of *n* = 5, field-cooling (*∆T*), seems to be roughly saturated to ~ 200 K in all the pump fluences. It is interesting to note that ∆*T* increases with respect to the pump fluence for the case of *n* = 15 (30 K for 9.9 mJ cm^−2^ and 244 K for 16.5 mJ cm^−2^ fluence). With increasing the pump fluence and thus, providing much an excessive energy to the system, the existence of the MCE was observed even in the case of *n* = 15, indicating that the ultrafast MCE on a femtosecond timescale could be a universal phenomenon.

In the context of the 3TM, the shortened *t*_eq_ under an external field should be directly related to the increase of channel widths (*G*_el_, *G*_es_ and *G*_ls_). One may consider that both *G*_ls_ and *G*_es_ might be modified by an external field. A series of 3TM fittings to the data with *G*_ls_ and *G*_es_ being free parameters has been performed. Through fittings, we note that with increasing **H**, the fitting value of *G*_es_ decreases, which might explain the experimental observation of *∆T*_s_ *;* On the other hand, decreased *G*_es_ values cannot explain the trend of *t*_eq_ with respect to **H**, because a decreased *G*_es_ would result in a longer *t*_eq_, which is contradictory to the experimental observation. Therefore, only a possible option is that only *G*_ls_ changes with **H**. In Fig. 4a, the field-dependent *G*_ls_ normalized by *G*_ls_ at **H** = 0 (*G*_ls_ */G*_ls(**H**=*0*)_) together with *∆T*_s_ for *n* = 5 and 15 sample is plotted. In the case of *n* = 15, relatively small modification of *G*_ls_ was observed for *θ*_H_ = 23°. *G*_ls_ */G*_ls(**H**=*0*)_ ~ 1.2 at **H** = 1.70 kOe as shown in Fig. 4a. On the other hand, in the case of *n* = 5, *G*_ls_ is ~4 × 10^16^ W m^−3^ K at **H** = 0 Oe and increases substantially to be saturated at 2.2 × 10^17^ W m^−3^ K for **H** = 0.90 kOe, above which *G*_ls_ remains unchanged, as shown in the inset of the figure. *G*_ls_ */G*_ls(**H**=0)_ ~ 5.5 at **H** = 1.70 kOe. Note that the saturation field of *G*_ls_ is similar to the saturation field of the loop in Fig. 2b. Similarly, for *n* = 10, *G*_ls_ is ~1.2 × 10^17^ W m^−3^ K at **H** = 0 Oe and increases to a saturation of 6.0 × 10^17^ W m^−3^ K at **H** = 0.60 kOe similar to saturation field of the hysteresis loop in Fig. 2b, leading to *G*_ls_ */G*_ls(**H**=0)_ ~ 5.0. Note the striking similarity in the trend between *G*_ls_ */G*_ls(**H**=0)_ and *∆T*_s_, suggesting that the field-cooling of spin temperature (*∆T*_s_) is involved directly in the modification of *G*_ls_ under an external magnetic field. The modification of *G*_ls_ by the external field is phenomenologically linked to the ultrafast MCE, which is expected from the fact that the extra energy pumped by the laser pulse should be dissipated eventually into the lattice, while the spin temperature is affected by the external magnetic field. The effective modification of energy paths (*G*_el_, *G*_es_ and *G*_ls_) is not new. In the case of semiconductor, where the 2TM model (electron and lattice temperatures) has been effectively applied, the modification of *G*_el_ by pump fluence has been reported^35–37^. The field-dependent lattice–spin and electron–spin interaction have recently been reported for molecules composed of transition metals^38, 39^. The modified *G*_ls_ should modify the temperatures of the spin and lattice. If *G*_ls_ becomes larger under an external field, the lattice will absorb the heat in the spin system more rapidly, leading to a larger MCE.

The remagnetization curve after the maximal demagnetization was fitted for *t* < 10 ps with an exponential function. The exponential fitting with ${A_0}{{\rm e}^{ - \frac{t}{{{\tau _{{\rm{eff}}}}}}}}$ for the initial remagnetization is used widely^40, 41^, where *A*_0_ is approximately proportional to (*T*_s_^max^ *−T*_eq_) and *τ*_eff_ is an effective relaxation time. In particular, it was reported that *τ*_eff_ may be a direct measure of an effective characteristic time of the lattice–spin interaction^40–43^. As discussed above, *t*_eq_ is shortened significantly in the case of *n* = 5. A smaller *t*_eq_ due to the ultrafast magnetic cooling under the field will result in a smaller *τ*_eff_ as well. Indeed, in the case of *n* = 5, *τ*_eff_ decreases with increasing **H**, as shown in Fig. 4b, consistently confirming the MCE as analyzed based on 3TM.

The 3TM calculation suggests that, although *t*_eq_ becomes shorter under **H**, *T*_eq_ remains the same, irrespective of **H**. Therefore, it is expected that *A*_0_ should become smaller with increasing magnetic field, because *T*_s_^max^ is cooled under the field. A decrease in *A*_0_ with respect to the field is observed for *n* = 5 as shown in Fig. 4c. For *n* = 15, no significant change in *G*_ls_ and thus, no substantial MCE is observed according to the 3TM analysis, suggesting that *A*_0_ is constant, as confirmed in Fig. 4c. In the case of *n* = 5, both *A*_0_ and *τ*_eff_ at a **H** = 0 field reduces to almost half at a field higher than 0.60 kOe. Considering that *A*_0_ is related to the degree of spin temperature cooling and *τ*_eff_ is also related to *t*_eq_, the contributions from both parameters should be included in *G*_ls_.

The field dependence of *τ*_eff_ has already been observed experimentally for a semiconductor system^44^. In the semiconductor, the contribution from *T*_e_ is negligible and *τ*_eff_ is approximated to be the characteristic relaxation time (*τ*_ls_) of the lattice-spin interaction channel, where *τ*_ls_ ~ *C*_s_/*G*_ls_^42, 43^. It was reported for such a system that the *C*_s_ value is proportional to the external magnetic field^45, 46^. Therefore, the decrease in *τ*_*ls*_ under the field was attributed to the increased *G*_ls_.

It should be mentioned that the ratio *G*_ls_ */G*_ls(**H**__=__0)_ is believed to represent the variation of the channel width of energy transfer under external magnetic field between the spin and the lattice. The dissipated heat from spin should be better transferred to lattice with a larger channel width under external magnetic fields. We have carried out experiment for different pump fluences of 9.9 to 16.5 mJ cm^−2^ and analyzed with 3TM model. Interestingly, although *G*_ls_ as well as *G*_es_ and *G*_el_ is effectively reduced with respect to the fluence as in Table 1, there seems to be no significant correlation between the ratio *G*_ls_ */G*_ls(**H**__=__0)_ and the fluence as in Fig. 4d, implying that the ratio could be a practically good parameter reflecting the effective channel width variation by the magnetic field, irrespective of the fluence variation. If the direct fundamental channel between the spin and the lattice is only related to the spin–orbit coupling, field-dependent varying quantities such as magnetostriction related to the spin–orbit coupling might play a role in modifying the *G*_ls_^47, 48^. We encourage further theoretical works regarding the variation of the energy transfer channel width under an external magnetic field.

As seen in Fig. 4e, with proper tuning of external parameters such as the pump fluence and the external field direction, the MCE was found to be observable. The giant ultrafast MCE by cooling the spin temperature up to 200 K is clearly manifested at the fluence of 16.5 mJ cm^−2^, implying that the MCE by the forced spin alignment could be universal phenomenon even on a sub-ps timescale. The observed ultrafast giant MCE opens a new possibility in engineering the mechanism of heating and energy transfer on a femtosecond timescale. Controlled MCE behavior by an external magnetic field, combined with the easy tunability of *G*_ls_ and other magnetic properties for the nanostructured Co/Pt multilayer, will be particularly useful and promising for spintronic applications with devices operating at an ultrafast speed, where the management of heat and energy transfer becomes increasingly important.

In summary, the dynamics of time-resolved hysteresis loop in Co/Pt multilayers were investigated systematically with a repeat number n of 5, 10 and 15. Pump-beam modulated TR-MOKE measurements were analyzed comprehensively using 3TM. From an experimental demonstration of the existence of giant MCE on an ultrafast time scale, it was found that MCE can be controlled via effective manipulation of the lattice–spin interaction through modification of multilayer parameter and pump fluence under an external field. This study is not only a proof-of-principle experiment but might provide the possibility of future applications utilizing the femtosecond and/or picoseconds magnetic cooling phenomenon, opening a door for studies and applications of ultrafast magnetic cooling on a sub-picosecond timescale.

## Methods

### MOKE measurement

Time-resolved magneto-optical Kerr effect (TR-MOKE) measurements in a pump-probe geometry (Supplementary Fig. 1) were carried out for Co/Pt multilayers. Mechanical chopping modulation at 750 Hz was implemented in a pump-beam path, combined with a lock-in amplifier to discriminate purely pump-induced magnetization changes to improve the signal-to-noise ratio. The measurement at 44 Hz modulating in synchronization of pump-probe stroboscopic frequency with the field-cycling frequency was also carried out to confirm the result at 750 Hz modulation. TR-MOKE signal with the probe-beam modulation was also taken. The pump pulses were generated by a Ti:sapphire multipass amplifier operating at 3 kHz repetition rate with a center wavelength of 780 nm and a pulse duration of 25 fs. The probe pulses with the same wavelength were generated by a beam splitter. The Wollaston polarizer was positioned to split out the s- and the p-polarization component in front of the two photodiodes, each of which detects the s- and p-polarization component, respectively. This measurement leads to a difference between the s- and p-polarization components of the probe pulses modified by MOKE at the reflection off a film surface. The pump fluence was varied from 9.9 to 16.5 mJ cm^−2^ and the probe fluence was 0.3 mJ cm^−2^. The delay of line was implemented at the pump-beam line with a delay time up to 700 ps. For TR-MOKE measurement, an external field was applied with an angle of 0 and 23° to the surface normal of the sample, and swept up to 1.70 kOe, to measure the MOKE hysteresis loops at each delay. The external magnetic field was cycled 15 times to produce the averaged TR-MOKE hysteresis loop at each delay. The time step of each delay was 50 fs.

### Samples

[Co(6.2 Å)/Pt(7.7 Å)]_*n*_ multilayer films with *n* = 5, 10 and 15 were deposited by dc magnetron sputtering on Si substrates, and capped by a 22 Å Pt to protect the oxidation of the surface. The structure of the Co/Pt multilayers with well-defined interfaces was confirmed by a low angle X-ray diffraction and the extended X-ray absorption fine structure analysis. All the films exhibited PMA and typical saturation magnetization values^19–23^, as confirmed by a vibrating sample magnetometer (VSM).

### Data availability

The data that support the findings of this study are available from the corresponding authors upon reasonable request.

## Electronic supplementary material

Supplementary Information
