# Supplementary material for: Ultrafast giant magnetic cooling effect in ferromagnetic Co/Pt multilayers
Source: Nat Commun. 2017 Oct 6;8:796. doi: 10.1038/s41467-017-00816-w (PMC5630601; doi:10.1038/s41467-017-00816-w)
Supplement: Supplementary file 1 — Supplementary Information [file 41467_2017_816_MOESM1_ESM.pdf]

Supplementary Note 1. Optical layout for the TR-MOKE measurement setup

A commercial CEP (carrier envelope phase)-stabilized multipass amplifier laser (Femtopower, Femtolaser. Inc.) was used as the light source for TR-MOKE. The laser was operated at a 780 nm wavelength, 3 kHz repetition rate, and 25 fs pulse width. A pair of BK7 prisms were used to adjust the group velocity dispersion of the amplifier output. A 50:50 pellicle beam splitter was used to split the output to generate the pump and probe beams. Both the pump and probe beams were set to have s-polarization. The intensities of the pump and probe beams were adjusted by a half wave-plate together with a linear polarizer. The pump beam was focused on the sample at a normal incidence. The mixing angle between the pump and the probe beam was set to 35°. The angle of the magnetic field with respect to the pump beam direction was varied from 23° to 67°. The reflected probe pulses from the sample surface were sent to the Wollaston polarizer to detect the s- and p-polarized outputs using two Si photo-diodes of PD(1) and PD(2) in the figure. For the reflectance measurements, the reflected probe beam was measured directly using the PD(3) to record its intensity change. The time resolution from the auto-correlation measurement was 200 fs or less, which is the result of the phase-front tilt at the maximum mixing angle of 67°.

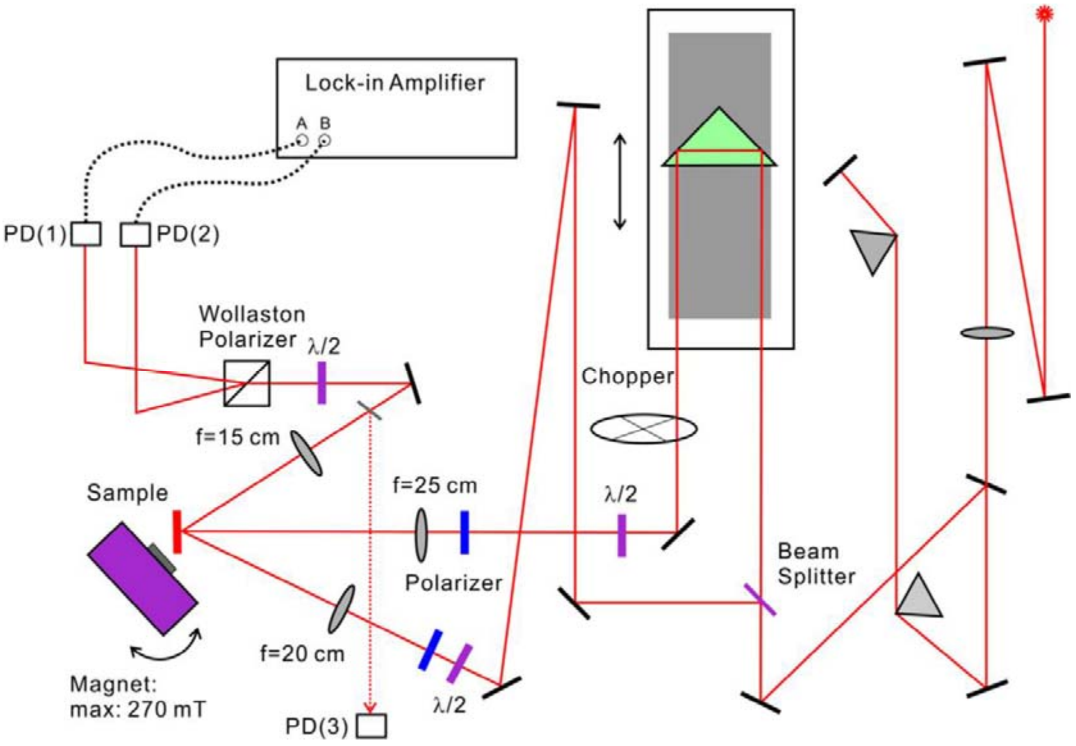

Supplementary Figure 1. **Schematic of TR-MOKE setup.**

Supplementary Note 2. Comparison of static and TR-MOKE hysteresis loops

We compared two loops measured by TR-MOKE with a pump-beam modulation at t = 700 ps (red open circle) and static MOKE (black open square) measurement without pump-beam, as shown in Supplementary Figure 2. The static MOKE hysteresis loops were found to be the same as the loop measured by VSM, where the loop shape variation with respect to n was also as expected from previous reports[1-5]. Interestingly, for n = 5, the TR- and static MOKE loops were different, which is a typical aspect of the "irreversible" process observed frequently in the stroboscopic pump-probe experiment[6, 7]. The irreversible trend appears to be weakened as n increases. In the case of n = 15, the two loops are similar, but a detectable difference in the coercive field exists between the static MOKE and stroboscopic TR-MOKE loops.

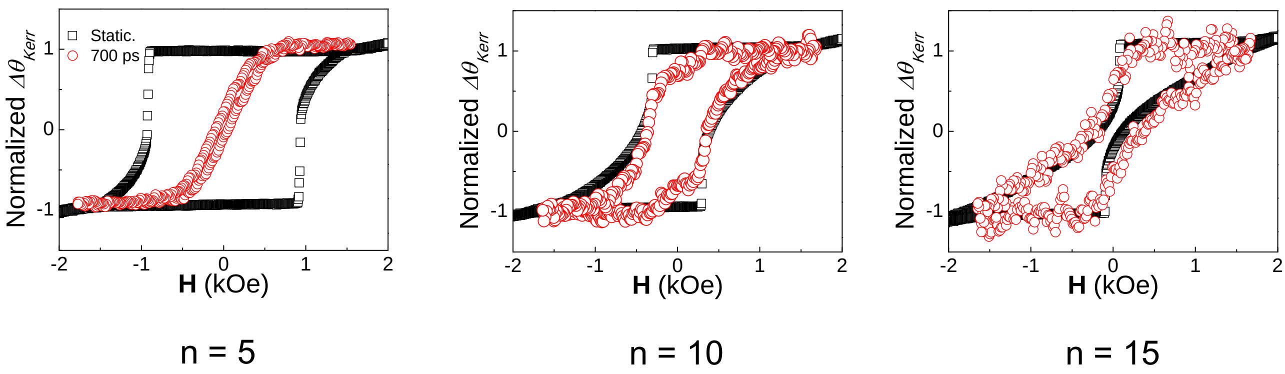

Supplementary Figure 2. Comparison of static MOKE (black open square) and TR-MOKE hysteresis loops (red open circle) at t = 700 ps.

Supplementary Note 3. Comparison of the hysteresis loops with a pump-beam and a probe-beam modulation

The hysteresis loops measured with a pump-beam and probe-beam modulations were also compared, as demonstrated in Supplementary Figure 3. In Supplementary Figure 3a, the TR-MOKE hysteresis loops with a pump-beam modulation for t = 0.3, 1, 10, 31, 293, and 700 ps, where a hysteresis loop height decreases in all cases after showing a maximum at t = 300 fs. On the other hand, as in Supplementary Figure 3b, TR-MOKE hysteresis loops with a probe-beam modulation exhibits an opposite trend, where a hysteresis loop height increases in all cases of n after t = 300 fs. The contrasting difference is well understood. Because the probe-beam modulated TR-MOKE signal is proportional to the sample magnetization, M, the loop height should increase with respect to time during remagnetization after the maximum demagnetization at t = 300 fs, as shown clearly in Supplementary Figure 3b. For the pump-beam modulated the TR-MOKE case, however, the signal was proportional only to the change in magnetization, ΔM, induced by the pump-beam is detected so that the induced magnetization change becomes smaller after the initial maximum demagnetization at t = 300 fs, as shown in Supplementary Figure 3a.

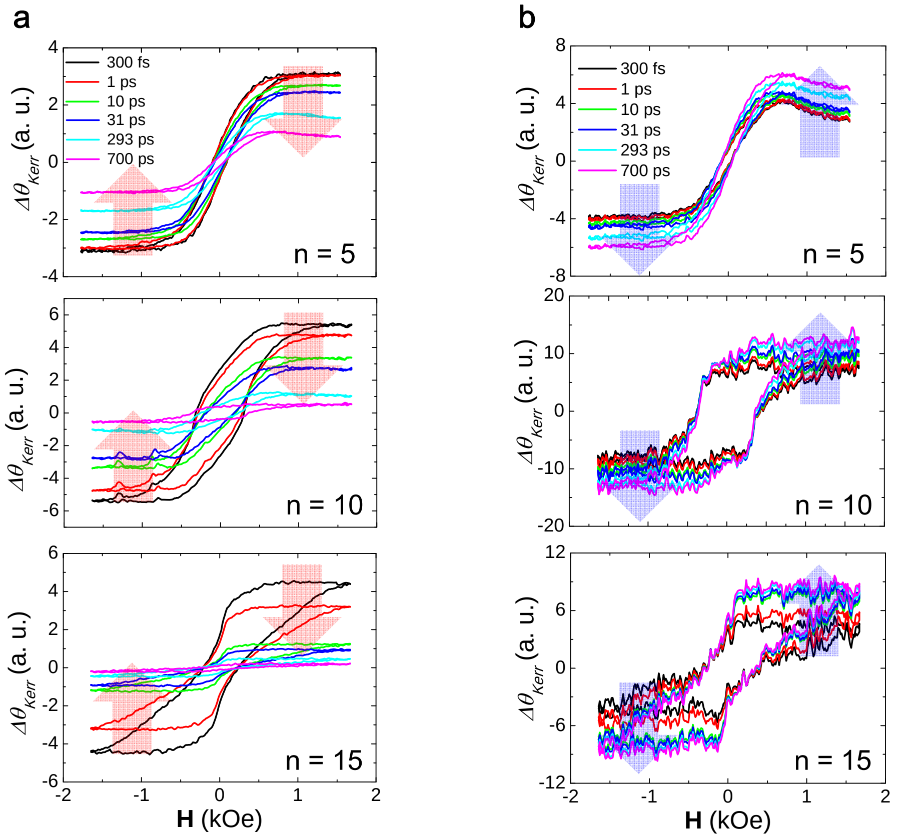

Supplementary Figure 3. TR-MOKE hysteresis loops for n = 5, 10, and 15 at t = 0.3, 1, 10, 31, 293, and 700 ps. The arrows are guide showing the trend with time. (a) The pump beam modulation Kerr hysteresis loop. (b) The probe beam modulation Kerr hysteresis loop.

Supplementary Note 4. Irreversible feature with variation of pump fluence

We have carried out a series of experiments to compare the loops measured by modulating the probe beam at a negative time (before the time zero) with a static loop. The pump laser fluence has been also varied from 9.9 to 16.5 mJ cm^{-2}. The results are shown in Supplementary Figure 4.

The significant change of the loop shape in terms of the width is observed for different fluences for the case of n = 5, while the loop height is observed to be the same, implying that the full saturation has been achieved for a given field strength in all the cases of the present study. The irreversible feature of the stroboscopy is clearly evidenced by obviously narrower widths with respect to the higher fluences. For the case of n = 15, no significant change is observed between the static and the pumped loop at a negative time. With a closer look, there still exists a little reduction of the loop width with the increase of fluence.

We have further carried out experiments to measure probe-beam modulated hysteresis at various delays as seen in Supplementary Figure 5. It should be reminded that the probe-beam modulated TR-MOKE measures a signal proportional to M itself, while the pump-beam modulated TR-MOKE measures a signal proportional to ΔM. From t = -1.0 ps to +1.2 ps, the loop shape is observed to decrease, while the loop height is drastically reduced after t = 0 due to the demagnetization by the pump. The H_c value in the probe-beam modulated hysteresis remains to be almost the same for both n = 5 and n = 10 cases within the error, which is not like the case of H_c^{pump}. We have also varied the fluence from 9.9 to 13.2 mJ cm^{-2}. With an increase of fluence, H_c obviously decreases. The decrease of H_c with an increase of fluence is also associated with irreversible feature.

The analysis of ΔM based on the pump-modulated TR-MOKE signal is based on the assumption that the $\Delta M(t) + M(t)$ remains the same at any time; in other words, the magnetization information on an ultrafast timescale is also reflected in the pump-induced magnetization change without any loss. We have checked whether the sum is indeed invariant along the time delays. Examples of added loop with a proper linear sum of the pump-beam modulated (~ ΔM) and the probe-beam modulated TR-MOKE signal (~ M) are plotted in Supplementary Figure 5. For the case of n = 15, pump-beam modulated TR-MOKE signal is added to the probe-beam modulated TR-MOKE signal at each delay. In addition process, the pump-beam modulated TR-MOKE signal is multiplied by a factor of $-\frac{1}{15}$, where the factor is determined by the lock-in amplifier configuration in the measurement and found to be the same for all the time delays. The invariance of the summed hysteresis loops at all the negative and positive time delays is confirmed. Examples of summed hysteresis loops of multilayers with n = 5 and 15 at a fluence of 13.2 mJ cm^{-2} for t = -0.4, 0.0, and +0.4 ps are plotted in the Supplementary Figure 6.

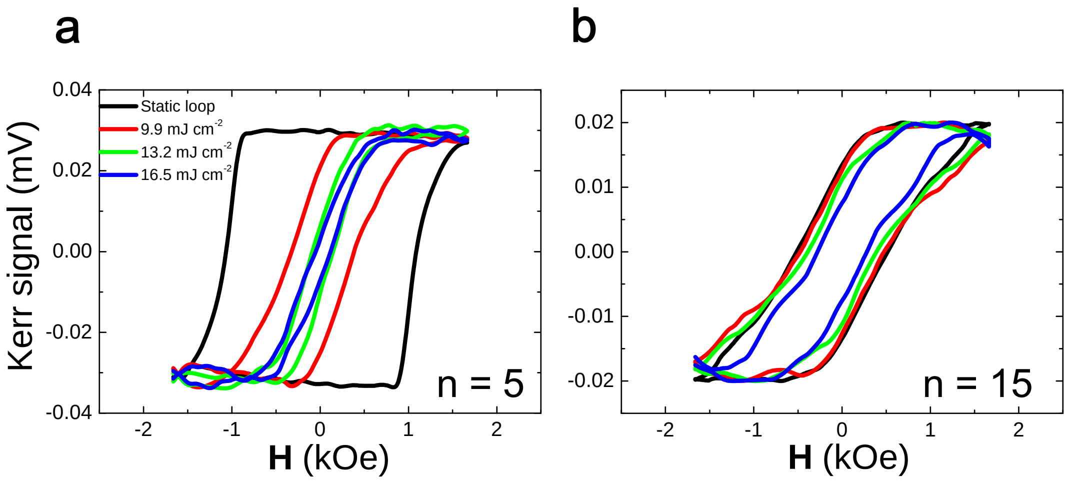

Supplementary Figure 4. Kerr hysteresis loops at *t* = - 20 ps under different fluences (red, green and blue) and static loop (black line). (a) Kerr hysteresis loop in $[Co/Pt]_5$ multilayer sample. (b) Kerr hysteresis loop in $[Co/Pt]_{15}$ multilayer sample.

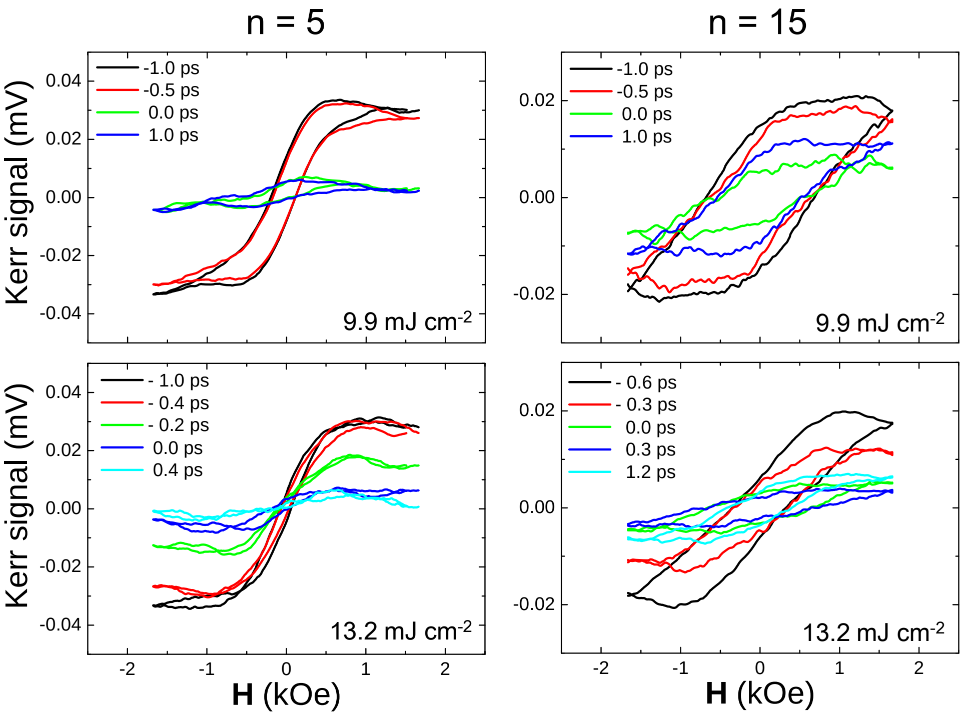

Supplementary Figure 5. Probe-beam modulated hysteresis with fluences of 9.9 (top row) and 13.2 mJ cm^{-2} (bottom row) for n = 5 and 15 multilayer.

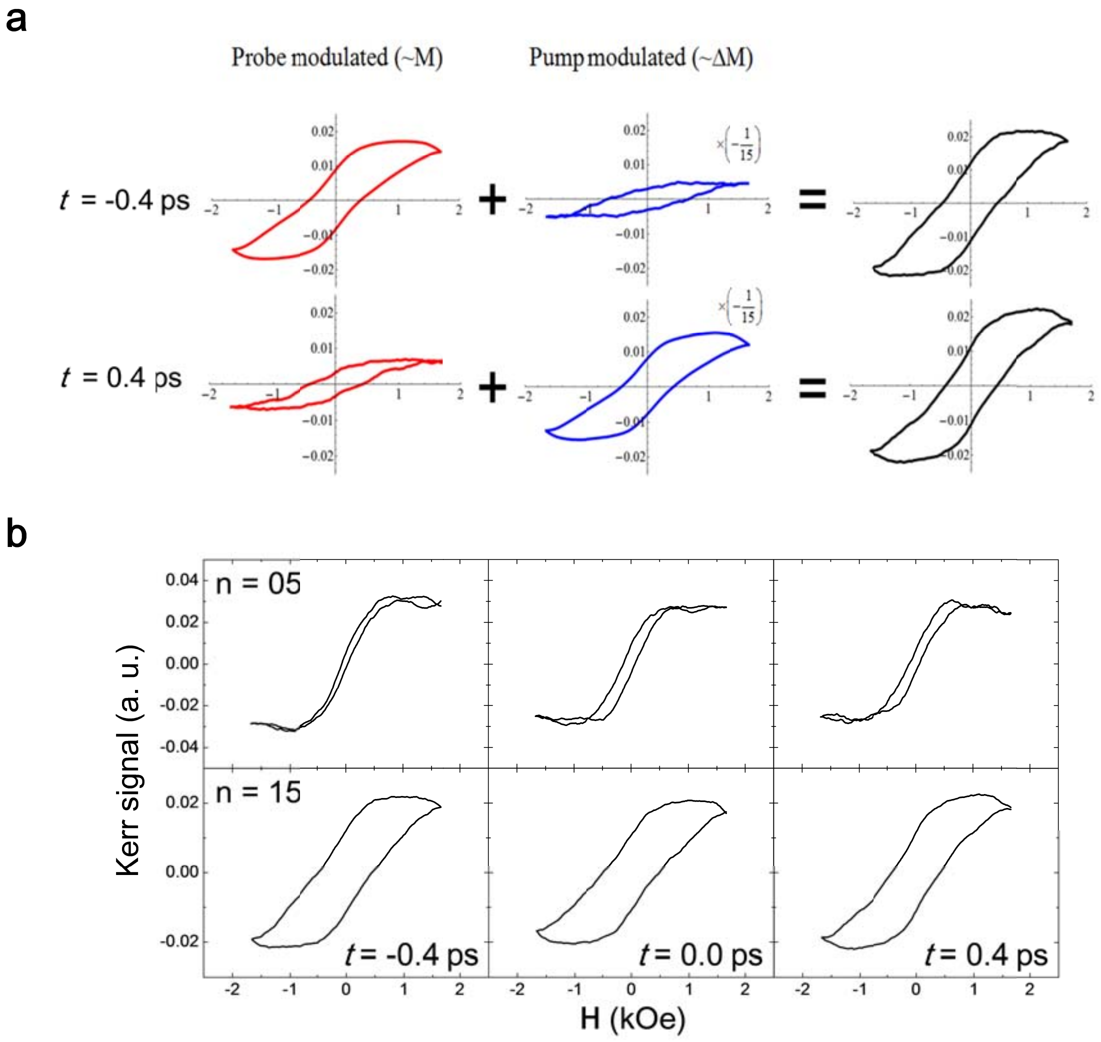

Supplementary Figure 6. Check the conservation of summed magnetization of $\Delta M + M$ at each delay time (a) Schematic diagram of procedure of $\Delta M + M$ at t = -0.4 and +0.4 ps for n = 15. (b) Summed hysteresis loop of pump- and probe-beam modulated hysteresis for n = 5 and 15 with fluence of 13.2 mJ cm^{-2}.

Supplementary Note 5. Synchronization of pump-probe and external field frequency

We have confirmed that the observed TR-MOKE result measured with 750 Hz chopper and lock-in amplifier configuration remains invariant although we change the stroboscopy frequency synchronized with field-cycling frequency of 44 Hz, as demonstrated in Supplementary Figure 7. To confirm that the observed TR-MOKE signal and its trend is not originated from the irreversible initial state discussed above, we synchronized all the frequencies of pump-probe stroboscopy, mechanical chopper, and external field cycling, where we could exclude the possibility of the irreversible initial state effect since the TR-MOKE signal is measured with a full saturation of the sample at every stroboscopic cycle. We have built a capacitance arrays of 6.6 μF to guarantee the impedance matching and the sufficient supply of a current to the electromagnet. Air-cooling of the capacitance array was extremely important to prevent the occasional explosion of capacitances. Cooling an electromagnet efficiently by means of water jacket was also essential to have a stable current at AC field-cycling. In the end, the synchronization of TR-MOKE stroboscopy and the external field-cycling at 44 Hz with the maximum field strength of 2.3 kOe was achieved. In a synchronized setup, specific time delays correspond to different field values. Different field values are controlled by shifting the relative phase of field-sweeping to the pump signal.

The examples of pump-beam modulated TR-MOKE signal at various relative phases (or corresponding field strengths) are plotted in Supplementary Figure 7a for the case of n = 5. The measured signal is proportional to ΔM. It is observed that the overall TR-MOKE behavior was found to be almost the same as in the case of the results from the unsynchronized setup with 750 Hz chopper frequency, for all n cases. The comparison of the pump-induced TR-MOKE results between the cases of the synchronized and the unsynchronized setup is plotted in Supplementary Figure 7b for n = 5. It is clearly shown that the TR-MOKE signal behavior is the same within the measurement error range. Based on these results, we claim that the analysis of the MCE behavior based on the TR-MOKE from the unsynchronized setup is valid.

We have also checked the probe-beam modulated hysteresis loop under synchronization at 44 Hz to measure the signal directly proportional to the magnetization (M) during the stroboscopic measurement, as in Supplementary Figure 7c. The loop with a pump-beam totally blocked, corresponding to the case of the static loop or loop at the negative time delay, is plotted together with the loop at a pump-beam fluence of 13.2 mJ cm^{-2} for the case of n = 5. Although the loop clearly exhibits the irreversible behavior, it should be noted that the saturated magnetizations are at the same level for the two cases within the measurement error, implying the observed pump-beam modulated TR-MOKE is indeed representing the amount of magnetization change during the stroboscopic measurement since the sample is fully saturated during the field- and the stroboscopic cycling. Thus we consider that the new results concretely support

our analysis of the MCE under applying the saturating field. The normalized TR-MOKE from the synchronized setup for n = 5 and n = 15 is plotted in Supplementary Figure 7d for different fields, where a clear sign of MCE is again observed under **H** = 2.3 kOe compared to the case of **H** = 0 Oe.

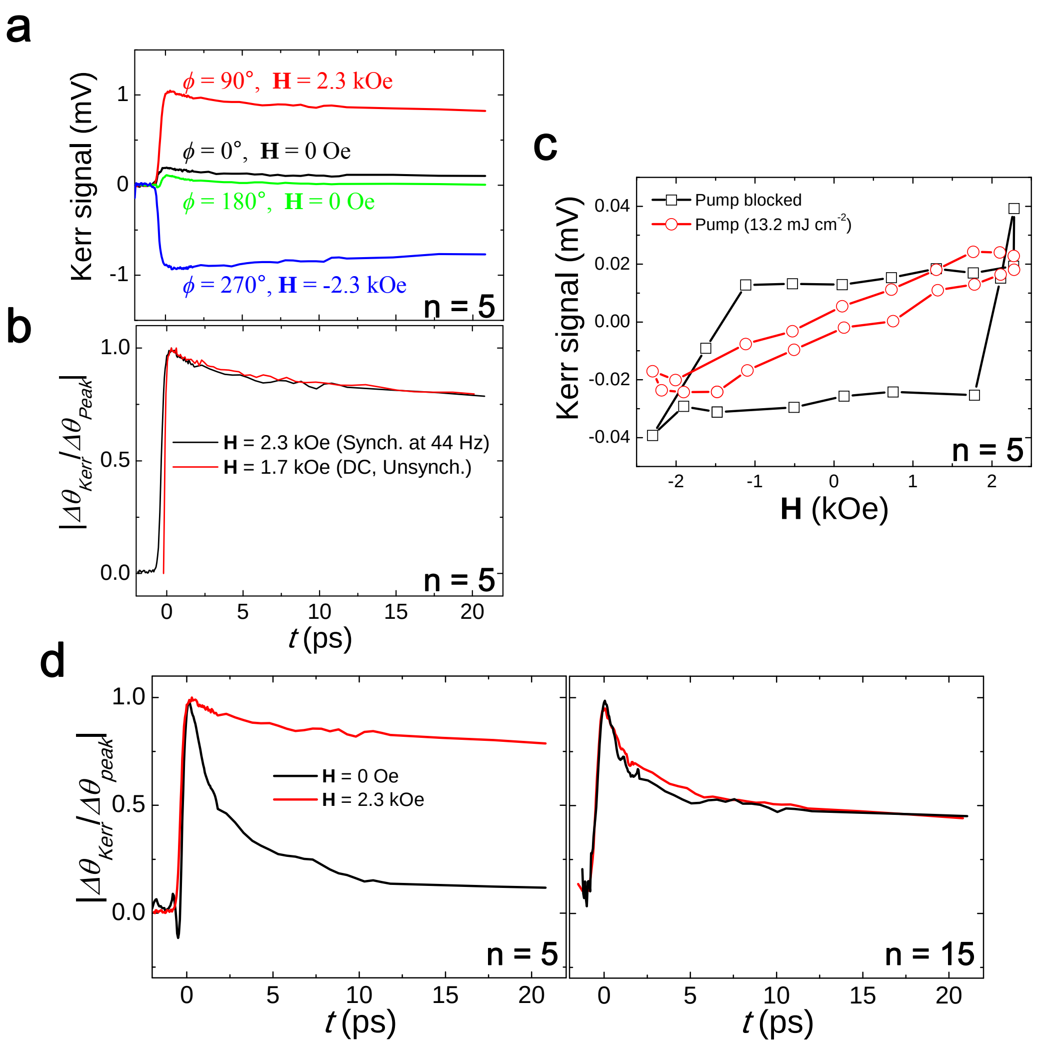

Supplementary Figure 7. Result of synchronized TR-MOKE signal. (a) Pump-beam modulated TR-MOKE signal (~*ΔM*) synchronized with different relative phases (or corresponding different field values) of an external magnetic field. (b) Comparison between synchronized and unsynchronized pump-beam modulated TR-MOKE signals. (c) Hysteresis loop of synchronously probe-beam modulated TR-MOKE signal (∝ ***M***) with no fluence (pump-beam blocked) and a fluence of 13.2 mJ cm^{-2} at t = -20 ps. (d) Normalized, synchronized pump-modulated TR-MOKE signal under fields of 0 and 2.3 kOe for n = 5 and 15.

Supplementary Note 6. Validity of 3TM fitting

First, the two temperature model (2TM) was applied to fit the TR-MOKE signals at **H** = 0 with a conventional assumption that a spin temperature promptly follows an electron temperature[8-12]. The 2TM equation is then simplified as

$$\begin{aligned} C_{e0}T_e(t)\frac{dT_e}{dt} &= G_{el}\left(T_l(t)-T_e(t)\right)+P(t) \\ C_l\frac{dT_l}{dt} &= G_{el}\left(T_e(t)-T_l(t)\right) \end{aligned}, \tag{1}$$

where the thermal diffusion term is neglected. For convenience, the following Supplementary Equation 2 was used to account for a real MOKE signal that was modulated by the pump beam to give a zero background signal before time zero.

$$S_M(t) = A\times\left[M\left(T_e(t)\right)-M(300\text{ K})\right], \tag{2}$$

where A is an arbitrary amplitude parameter. Numerical analysis was performed to check the effects of the parameters, C_{e0}, G_{el} and C_l. In the simulation, $P(t)$ was set to be a Gaussian function with a fluence of 7×10^{21} W m^{-3} and a pulse width of 200 fs. The 2TM was used to globally fit all the TR-MOKE signals under **H** = 0 Oe.

After achieving the parameters approximately by 2TM, the fit was extended to the TR-MOKE data using 3TM. The parameters fitted by 2TM for n = 5, 10 and 15 were used as the starting values of 3TM fitting under a range of magnetic fields.

Supplementary Figure 4 shows the results of comparison between the fittings by 2TM and 3TM for n = 5 and 10 under different fields. The 2TM fitting results appeared to fit the data well at **H** = 0 but exhibited a significant discrepancy at **H** = 1.70 kOe. In the case of n = 10, for example, the standard error of the regression by the 2TM at **H** = 0 for the initial 100 ps was 3.4, which was similar to the standard error by the 3TM fitting. At **H** = 1.70 kOe, however, the standard error by the 2TM became 6.3, whereas the standard error by the 3TM was approximately 1.5. In all cases, the 3TM clearly provided better fitting results than the 2TM, particularly for the case of non-zero external magnetic fields.

In previously reported low-temperature experiments, C_s was reported to be less than 10^4 J m^{-3}k^{-1} [13, 14]. The overall fitting in this study is not affected by a selection of the C_s value if $C_s < 10^4$ J m^{-3}K^{-1}. Therefore, a C_s value of 100 J m^{-3}K^{-1} was chosen in all the fits. In addition, the C_s values for several materials were quite low, as in the case of the fitting[13, 15]. Similarly, C_e was also set to be constant, irrespective of n because there is no reason for the effective electron number density around the Fermi energy to change substantially with n. Therefore, as in previous studies[11, 16, 17], C_e was just assumed to be

linearly proportional to T_e for all samples. On the other hand, the fitting suggests that C_l increases with n. This is understandable because the Co/Pt multilayer is considered to be a repeated superlattice structure[18], possibly generating a coherent phonon in an initial phase after laser pulse illumination. The increased C_l should lower the equilibrium temperature of spin, electron and lattice, considering that C_s is negligibly small and C_e does not depend on n.

The validity of the 3TM fitting was also checked by systematic variations of the field strength. The best 3TM fitting results of the TR-MOKE signals under a range of magnetic fields for n = 5, 10 and 15 were plotted together with the experimental data in Supplementary Figure 9, where excellent agreement between the experimental data and 3TM fittings were found in all cases of n and **H**.

The 3TM was also applied to analyze the TR-MOKE signal with variation of fluences as in Supplementary Figure 10, where the external field was applied normal to the films. As seen in the figure, 3TM is found to still fit the data well, where same values for heat capacities of spin, electron, and lattice are used with only freeing G_{el}, G_{es}, and G_{ls} parameters. The fitting results are listed in Table 1.

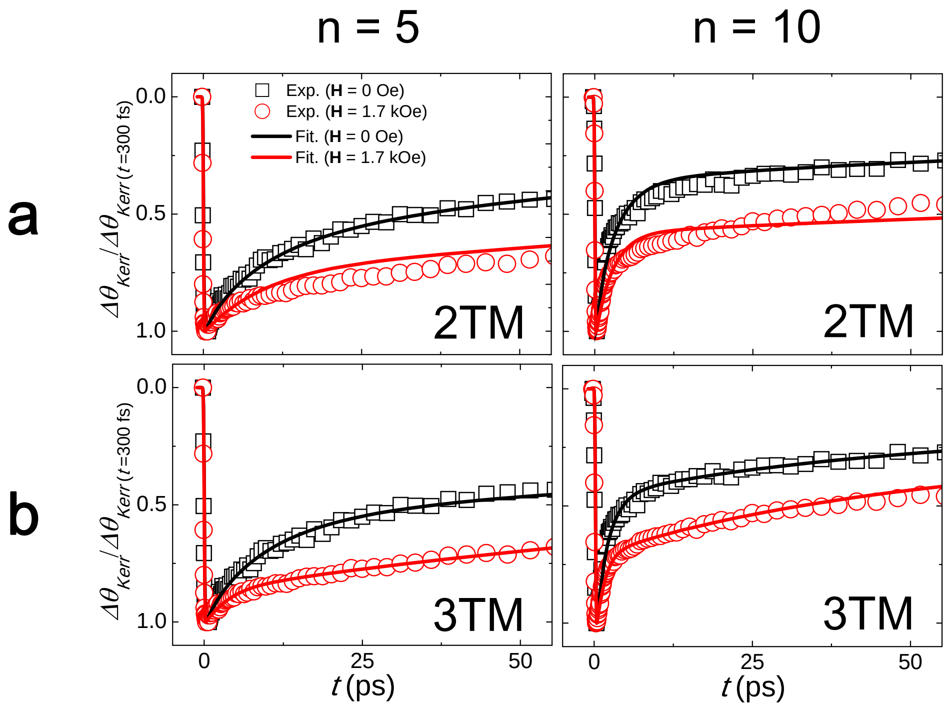

Supplementary Figure 8. Comparison of best fits of TR-MOKE signals using 2TM and 3TM for n = 5 and 10. (a) 2TM fitting results. (b) 3TM fitting results.

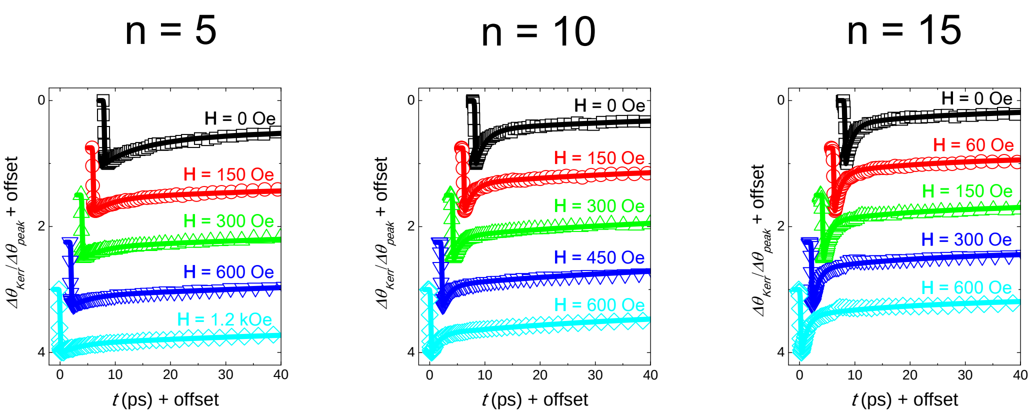

Supplementary Figure 9. **TR-MOKE signals (open) and fitting curves (solid lines) for n = 5, 10 and 15 under various external magnetic fields, as denoted in the graph. Arbitrary offset was taken for comparison.**

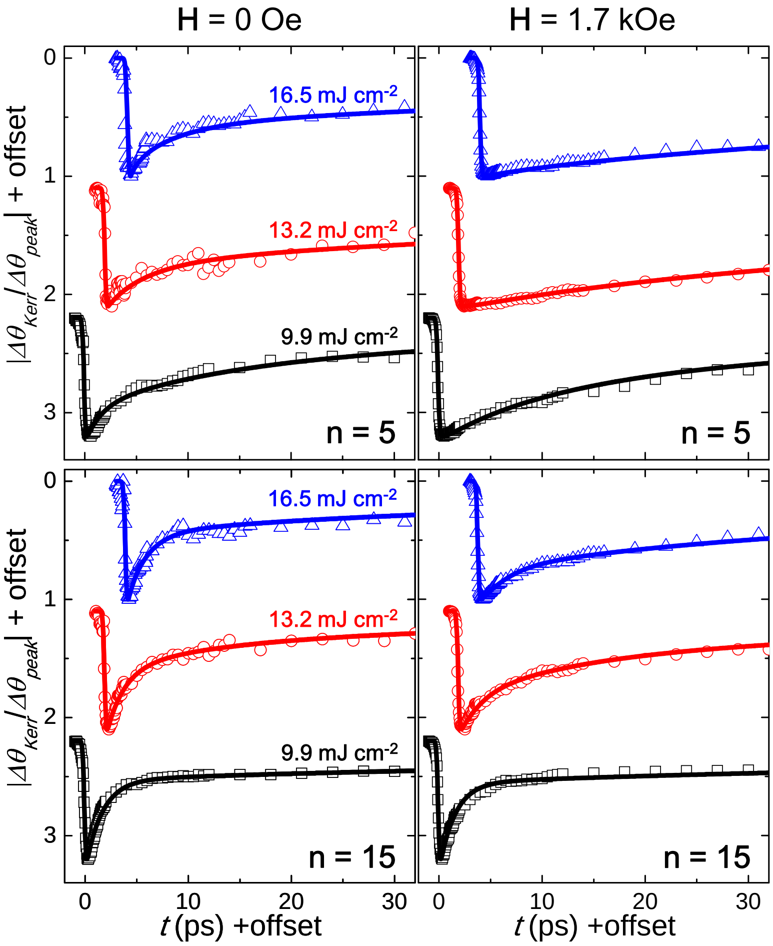

Supplementary Figure 10. TR-MOKE signal (open symbols) and 3TM fitting (lines) for various fluence at H = 0 and 1.7 kOe with θ_H = 0°.

Supplementary Note 7. Static MCE

We have carried out an experiment to determine the equilibrium MCE of Co/Pt multilayers. *M*-**H** curves of Co/Pt multilayers of n = 5, 10, and 15 at T = 300 ~ 360 K measured by SQUID are plotted in Supplementary Figure 11. The resulting temperature change is analyzed utilizing the Eq. (1) with effective total heat capacity determined by DSC (1.85 × 10^6 J $m^{-3}K^{-1}$ for n = 5 and 2.15 × 10^6 J $m^{-3}K^{-1}$ for n = 15) and by 3TM (1.63 × 10^6 J $m^{-3}K^{-1}$ for n = 5 and 3.19 × 10^6 J $m^{-3}K^{-1}$ for n = 15).

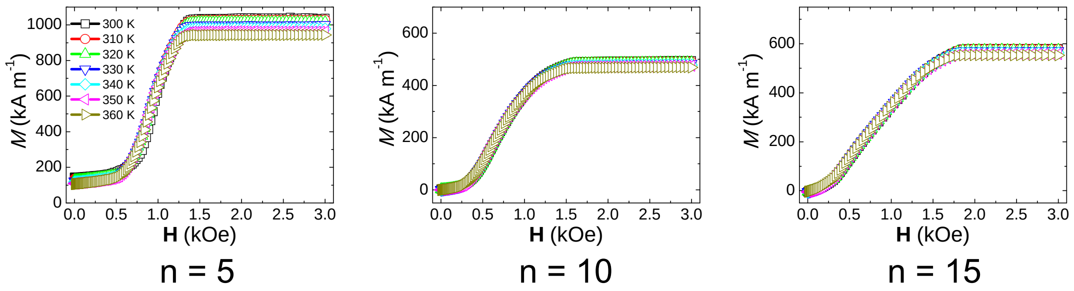

Supplementary Figure 11. Field dependent magnetization of $[Co/Pt]_n$ films with n = 5, 10, and 15, measured from 300 to 360 K.

Supplementary Note 8. Magnetic anisotropy measurement

The magnetic anisotropy was determined via analysis of the easy- and hard- axis hysteresis curves measured by vibrating sample magnetometer[19]. The results are shown in Supplementary Figure 12, where the effective anisotropy (K_{eff}) and anisotropy field ($\mathbf{H_k}$) for n = 5, 10, and 15 samples are plotted. Both K_{eff} and $\mathbf{H_k}$ decrease from 6.3 × 10^6 to 2.3 × 10^6 erg cm^{-3} and 12.6 to 6.6 kOe, respectively, with respect to n. $\mathbf{H_k}$ of all the samples are greater than 5 kOe, stronger compared to the external field strength (1.70 kOe). Thus, the external field applied along the film-normal direction could affect the sample responses more directly.

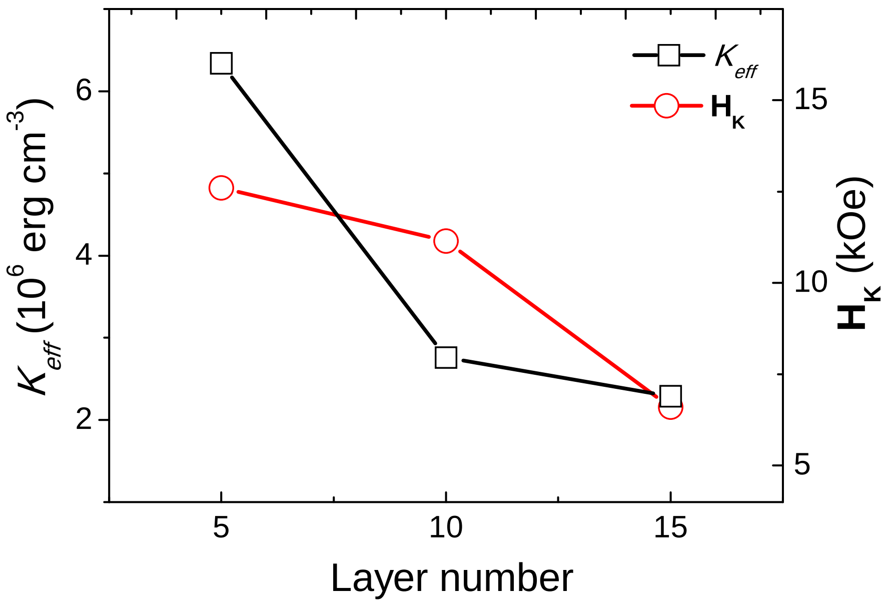

Supplementary Figure 12. Magnetic anisotropy constant (K_{eff}) and anisotropy field (H_k) for n = 5, 10, and 15.

Supplementary References

1. Hashimoto S., Ochiai Y., and Aso K. Ultrathin Co/Pt and Co/Pd multilayered films as magneto-o ptical recording materials. *J. Appl. Phys.* **67**, 2136-2142 (1990).

2. Hashimoto S., Ochiai Y., and Aso K. Fim thickness dependence of magneto-optical and magnetic properties in Co/Pt and Co/Pd multilayers. *J. Appl. Phys.* **67**, 4429-4431 (1990).

3. Zeper W. B., Kesteren H. W., Jacobs B. A. J., Spruit J. H. M., and Carcia P. F. Hysteresis, micros tructure, and magneto-optical recording in Co/Pt and Co/Pd multilayers. *J. Appl. Phys.* **70**, 2264-2271 (1991).

4. Yakushiji K., Saruya T., Kubota H., Fukushima A., Nagahama T., Yuasa S., and Ando K. Ultrath in Co/Pt and Co/Pd superlattice films for MgO-based perpendicular magnetic tunnel junctions. *A ppl. Phys. Lett.* **97**, 232508 (2010).

5. Zeper W. B., Greidanus F. J. A. M., Carcia P. F., and Fincher C. R. Perpendicular magnetic aniso tropy and magnetooptical Kerr effect of vapor deposited Co/Pt-layered structures. *J. Appl. Phys.* **65**, 4971-4975 (1989).

6. Roth T., Steil D., Hoffmann D., Bauer M., Cinchetti M., and Aeschlimann M. Dynamics of the c oercivity in ultrafast pump–probe experiments. *J. Phys. D* **41**, 164001 (2008).

7. Li S., Chen Z., Cheng C., Li J., Zhou S., and Lai T. Coercivity dynamics and origin of time-delay ed magneto-optical hysteresis loops in pump-probe Kerr spectroscopy. *J. Appl. Phys.* **113**, 05391 3 (2013).

8. Koopmans B., Malinowski G., Dalla Longa F., Steiauf D., Fähnle M., Roth T., Cinchetti M., and Aeschlimann M. Explaining the paradoxical diversity of ultrafast laser-induced demagnetization. *Nature Mater.* **9**, 259-265 (2010).

9. Atxitia U., Chubykalo-Fesenko O., Kazantseva N., Hinzke D., Nowak U., and Chantrell R. W. M icromagnetic modeling of laser-induced magnetization dynamics using the Landau-Lifshitz-Bloc h equation. *Appl. Phys. Lett.* **91**, 232507 (2007).

10. Atxitia U., Chubykalo-Fesenko O., Walowski J., Mann A., and Münzenberg M. Evidence for ther mal mechanisms in laser-induced femtosecond spin dynamics. *Phys. Rev. B* **81**, 174401 (2010).

11. Atxitia U. and Chubykalo-Fesenko O. Ultrafast magnetization dynamics rates within the Landau-Lifshitz-Bloch model. *Phys. Rev. B* **84**, 144414 (2010).

12. Bunce C., Wu J., Ju G., Lu B., Hinzke D., Kazantseva N., Nowak U., and Chantrell R. W. Laser-i nduced magnetization switching in films with perpendicular anisotropy: A comparison between measurements and a multi-macrospin model. *Phys. Rev. B* **81**, 174428 (2010).

13. Flokstra J., Gerritsma G. J., and Marel L. C. Spin-lattice relaxation in Potassium Chromium Alu m. *Physica B* **94**, 53-59 (1978).

14. Twardowski A., Swagten H.J.M., Jonge W. J. M., and Demianiuk M. Magnetic behavior of the diluted magnetic semiconductor $Zn_{1-x}Mn_xSe$. Phys. Rev. B **36**, 7013-7023 (1987).

15. Reeves M. E., Stupp S. E., Friedmann T. A., Slakey F., Ginsberg D. M., and Klein M. V. Field-d ependent specific heat of polycrystalline $YBa_2Cu_3O_{7-x}$. *Phys. Rev. B* **40**, 4573-4584 (1989).

16. Beaurepaire E., Merle J.-C., Daunois A., and Bigot J.-Y. Ultrafast Spin Dynamics in Ferromagnet ic Nickel. *Phys. Rev. Lett.* **76**, 4250-4253 (1996).

17. Bigot J.-Y. , Vomir M., Andrade L. H. F., Beaurepaire E. Ultrafast magnetization dynamics in fer romagnetic cobalt: The role of the anisotropy. *Chem. Phys.* **318**, 137-146 (2005).

18. Luckyanova M. N., Garg J., Esfarjani K., Jandl A., Bulsara M. T., Schmidt A. J., Minnich A. J., Chen S., Dresselhaus M. S., Ren Z., Fitzgerald E. A., and Chen G. Coherent Phonon Heat Conduction in Superlattices. *Science* **338**, 936-939 (2012).

19. Carcia P. F., Meinhaldt A. D., and Suna A. Perpendicular magnetic anisotropy in Pd/Co thin film layered structures. *Appl. Phys.Lett.* **47**, 178-180 (1985).
